# Top-Down Estimation of Particulate Matter Emissions from Extreme Tropical Peatland Fires Using Geostationary Satellite Fire Radiative Power Observations

**DOI:** 10.3390/s20247075

**Published:** 2020-12-10

**Authors:** Daniel Fisher, Martin J. Wooster, Weidong Xu, Gareth Thomas, Puji Lestari

**Affiliations:** 1 Leverhulme Centre for Wildfires, Environment and Society, Department of Geography, King’s College London, Aldwych, London WC2B 4BG, UK; weidong.xu@kcl.ac.uk; 2 NERC National Centre for Earth Observation (NCEO), Leicester LE1 7RH, UK; gareth.thomas@stfc.ac.uk; 3 RAL Space, STFC Rutherford Appleton Laboratory, Harwell Campus, Didcot OX11 0QX, UK; 4 Faculty of Civil and Environmental Engineering, ITB, JL. Ganesha No.10, Bandung 40132, Indonesia; pujilest@indo.net.id

**Keywords:** tropical peatlands, landscape fire, emissions, FRP, total particulate matter

## Abstract

Extreme fires in the peatlands of South East (SE) Asia are arguably the world’s greatest biomass burning events, resulting in some of the worst ambient air pollution ever recorded (PM_10_ > 3000 µg·m^−3^). The worst of these fires coincide with El Niño related droughts, and include huge areas of smouldering combustion that can persist for months. However, areas of flaming surface vegetation combustion atop peat are also seen, and we show that the largest of these latter fires appear to be the most radiant and intensely smoke-emitting areas of combustion present in such extreme fire episodes. Fire emissions inventories and early warning of the air quality impacts of landscape fire are increasingly based on the fire radiative power (FRP) approach to fire emissions estimation, including for these SE Asia peatland fires. “Top-down” methods estimate total particulate matter emissions directly from FRP observations using so-called “smoke emission coefficients” [*C_e_*; g·MJ^−1^], but currently no discrimination is made between fire types during such calculations. We show that for a subset of some of the most thermally radiant peatland fires seen during the 2015 El Niño, the most appropriate *C_e_* is around a factor of three lower than currently assumed (~16.8 ± 1.6 g·MJ^−1^ vs. 52.4 g·MJ^−1^). Analysis indicates that this difference stems from these highly radiant fires containing areas of substantial flaming combustion, which changes the amount of particulate matter emitted per unit of observable fire radiative heat release in comparison to more smouldering dominated events. We also show that even a single one of these most radiant fires is responsible for almost 10% of the overall particulate matter released during the 2015 fire event, highlighting the importance of this fire type to overall emission totals. Discriminating these different fires types in ways demonstrated herein should thus ultimately improve the accuracy of SE Asian fire emissions estimates derived using the FRP approach, and the air quality modelling which they support.

## 1. Introduction

**Figure 1:**
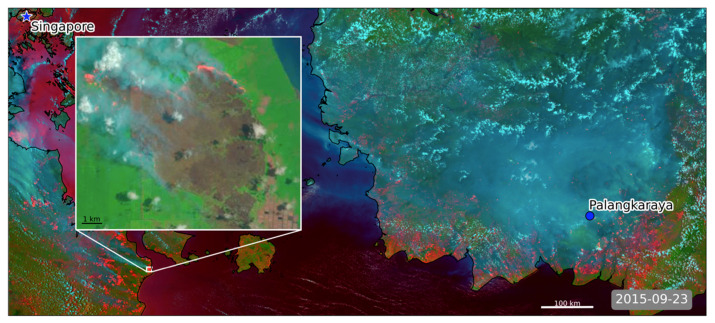
Satellite imagery of Kalimantan and South Sumatra taken on 23 September 2015 during the extreme landscape fires exacerbated by the strong El Niño-related drought. Main image: Visible Infrared Imaging Radiometer Suite (VIIRS) false colour composite (R: 3.7 µm; G: 0.865 µm; B: 0.488 µm) where clouds appear white, smoke blue—grey, and actively burning fires—bright red. Inset image: Landsat false colour composite (R: B6 (SWIR); G: B5 (NIR); B: B4 (VIS)) for the same day as the main image, focused on the longest of the Sumatran plumes present in the main image whose source region is boxed in white on the VIIRS imagery. Again, clouds appear white, smoke grey, and actively burning fires orange/red; also note the large burned area, which appears dark brown. The fire shown in the Landsat scene is plume/fire matchup Fire 7 in Figure 2.

In the second half of the 20th Century, changing land management practices in the lowland tropical swamp forests of equatorial South East (SE) Asia led many to be degraded from their natural forest-covered, persistently waterlogged state [1,2,3,4,5]. The carbon-rich peat surface now becomes dry enough to burn at times of low rainfall in many of these deforested and drained areas, leading to substantial increases in the region’s susceptibility to extreme landscape fire—particularly during the periodic droughts brought on by El Niño–Southern Oscillation events [6,7,8,9]. During the extreme 2015 El Niño, an overwhelming number of landscape fires occurred in the tropical peatlands of Kalimantan and Sumatra during September and October [10]. Truly extreme ambient atmospheric concentrations of particulate matter (PM) were generated across parts of Indonesia in particular, representing one of the worst sustained air pollution events ever recorded (see Figure 1, main image). PM_10_ concentrations sometimes exceeded 3000 µg·m^−3^, as reported in [10], and very substantial impacts on human mortality have been suggested as a result of this air pollution [11,12]. However, such estimates are built on as yet uncertain fire-emitted PM totals along with dose-response functions derived for Western populations that are generally subject to far lower, yet more long-term, PM exposures (e.g., those from urban air pollution).

Emissions from fires on peatlands and other landscapes are mostly derived using so-called “bottom-up” approaches, which usually require the combination of satellite-derived burned area data with information on fuel consumption per unit area. The latter can be difficult to obtain, especially perhaps where peatland fires burn down into the carbon-rich soil to varying degrees—generating fires that can persist in the smouldering phase for weeks or months and can consume very large amounts of biomass per unit area [13]. Such “bottom-up” approaches are also only able to be applied after the fire event has occurred, making them unsuitable to support near-real-time emissions estimates and air quality early warning applications [14,15]. An alternative fire emissions estimation approach, and one that can be applied in real-time, is the so-called “top-down” method linking satellite-derived fire radiative energy (FRE; MJ) observations directly to total particulate matter (TPM; g) emissions through a so-called “smoke emissions coefficient” [$C_{e}$; g·MJ^−1^]. This is possible as FRE is correlated with fuel mass combusted [16] and also removes the need to estimate fuel consumption per unit area, relying only on knowledge of an appropriate $C_{e}$ factor.

Tropical peat soils consist of ~54–60% carbon [17], and organic carbon is the primary particulate matter emission component resulting from its combustion [10]. Research foci related to peatland fire emissions have up to now primarily been focused on smouldering fires in this organic soil [8,18,19,20], which, whilst involving relatively slow combustion rates per unit area, produces substantially more PM emissions per unit of dry matter burned than do flaming or mixed phase (i.e., smouldering and flaming) fires in vegetation (e.g., [21]). These characteristics of smouldering peat are reflected in the TPM $C_{e}$ used to produce current “top-down” fire emissions estimates in peatland fires, these being far higher than those of tropical forest vegetation fires (see Section 2). However, it is currently unclear whether such high smoke emission coefficients are singularly appropriate for all fires occurring on tropical peatlands, because some areas may involve flaming as well as smouldering fuel consumption. Indeed, data collected by the Advanced Himawari Imager (AHI) carried onboard the Himawari-8 satellite, along with data from the Visible Infrared Imaging Radiometer Suite (VIIRS) and Landsat Advanced Land Imager (ALI) satellite instruments (Section 4.2 and Figure 1), show evidence of highly radiant peatland fires, which appear to involve substantial flaming combustion of surface vegetation in addition to smouldering peat [22]. The likelihood of smoke being injected above the atmospheric boundary layer during flaming combustion is greater than for smouldering combustion [23], and this in turn likely supports more significant transboundary transport of the polluted air (e.g., to Singapore and mainland Malaysia [24,25] in the region studied herein). Accurately identifying and estimating the PM emissions for such flaming peatland fires is therefore likely to be important for effective modelling of air pollution transport, and for better gauging their ultimate air quality impacts. The objectives of the current work are therefore to (i) examine the PM emission characteristics of more intensely radiant tropical peatland fires in particular, through the development and application of a more appropriate top-down TPM $C_{e}$ for such events, derived here using AHI fire radiative power (FRP; MW) products [26] and collocated observations of aerosol optical depth (AOD) obtained from VIIRS; and (ii) assess the impact of this updated $C_{e}$ compared to use of existing values from studies that have not considered discriminating between smouldering and mixed phase fires. Our ultimate aim is to use the findings from this work to improve real-time air pollution forecasting during peatland fire episodes, which we demonstrate include flaming combustion of surface vegetation as well as smouldering combustion of subsurface organic soils.

## 2. Landscape Fire Emission Estimation Overview

“Bottom-up” methodologies convert observations of terrestrial phenomena into estimates of atmospheric emissions using assumptions and/or additional terrestrial parameters, for example, fuel consumption per unit area measures [27,28]. The two most widely used “bottom-up” emissions inventories are the Global Fire Emissions Database (GFED) [28], and the Global Fire Assimilation System (GFAS) [27]. In both systems, information derived from Earth observation (EO) on either burned area (BA (m^2^); GFED) or FRP (GFAS) are converted into estimates of smoke emission via application of species-specific emission factors (EF; g·kg^−1^) applied to the bottom-up generated estimates of fuel consumption (kg). Fuel consumption estimates in GFED are produced by multiplying satellite-derived burned area measures by an assumed (modelled) fuel consumption per unit area, whereas in GFAS they are generated via application of biome-specific coefficients linking FRE and dry matter fuel consumption totals, which themselves have been generated using past comparisons of satellite-derived FRE data from GFAS and GFED fuel consumption values [27].

To support real-time air quality forecasting, we focus on an approach based on FRP measures, as they can be derived at the temporal resolution of the satellite rather than over multiple days as is typical of BA-based approaches [29]. Whilst significant advancements have been made during the last two decades in the active fire detection and fire characterisation algorithms that underlie FRP approaches (e.g., [29,30]), and landcover specific EF databases are becoming more detailed in their contents [31,32,33], there remain uncertainties in the FRP approach to fire emissions estimation. These can stem from (i) the relatively limited sampling frequency provided by the polar-orbiting satellites that currently dominate provision of FRP [34]; (ii) the fact that some of any surface-fire emitted FRP maybe intercepted by overlying tree canopies [35]; (iii) that some fires are too small or weakly burning to be detected in the most common spaceborne FRP data products (see comparisons by [36]); and (iv), in the case of tropical peatlands, from the rather limited (and until recently largely laboratory-derived) emission factors for peat burning [37]. Perhaps the most significant uncertainties are associated with the conversion between FRP and fuel consumption rate or totals. The conversion factor for this process has generally been derived in one of two ways. First, using measurements of FRP and fuel consumption in small laboratory-scale vegetation fires [16], although these may not be fully appropriate for application to satellite-derived FRP observations—especially in forested environments due to the issues outlined in [38], such as interception of radiated heat by forest canopies. Additionally, such conversion factors may not be applicable to types of fire involving at least some subsurface combustion [13], since fires burning underground will likely show a different amount of surface-emitted FRP per kg of fuel burned than do “normal” surface fires. Second, via comparisons between FRP data and GFED-derived fuel consumption totals (e.g., see [27]), which thus still leaves issues such as non-detection of smaller burned areas in GFED [39]—the under detection of peat burn area, in particular [40], and the use of difficult-to-estimate fuel loads [41] and combustion completeness [42] parameters within GFED. The last of these issues seems especially problematic for subsurface peat fires, where there remain rather limited measurements of peat depth-of-burn (DOB) [13], and where an accurate method to estimate the DOB of any particular satellite-detected fire remains lacking.

For these reasons, we focus on adapting the fully top-down “Fire Radiative Energy eMissions” (FREM) approach of [38,43] to tropical peatland fires. The FREM approach uses species- and biome-specific $C_{e}$ to directly link FRP data to the emissions of any particular species within the smoke, thus removing the need for the interim fuel consumption estimation step. The method is “top-down” as it relies on atmospheric and terrestrial remote sensing observations only, as detailed in [37,38,44,45]. In contrast to the Moderate Resolution Image Radiometer (MODIS) based approach of [44], the FREM approach also attempts to minimise the number of assumptions required when deriving each biome-specific $C_{e}$ by exploiting the almost continuous, very high temporal resolution FRP data available from geostationary sensors. Using these data, it is possible to derive an estimate of the total FRE released by a fire during the period when it produced a particular smoke plume. Each plume contains a certain amount of TPM, which itself can be estimated using satellite-derived AOD products [38,43,44,45] and further detailed in Section 3.3. Developing a set of matchup fires for which FRE and TPM values are determined enables the derivation of specific $C_{e}$ for the biome and fire-type of interest, and it is our aim here to do this exclusively for the type of more intensely radiant fires seen on tropical peatlands and shown in Figure 1.

## 3. Top-Down Estimation of Particulate Matter Emissions

### 3.1. Algorithm Requirements and Plume Digitisation

**Figure 2:**
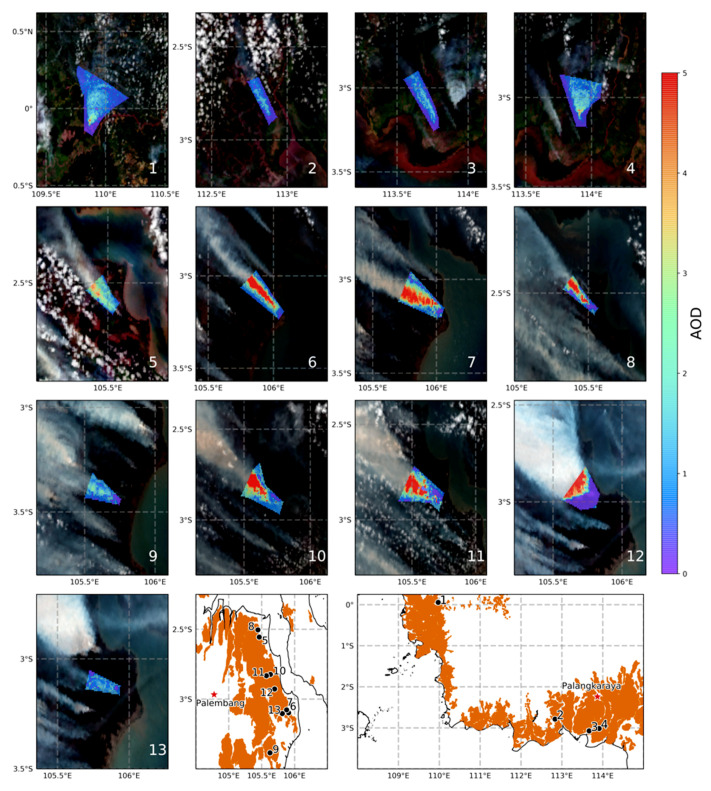
Plumes and overlain aerosol optical depth (AOD) from the thirteen highly radiant peatland fires on Sumatra and Kalimantan burning between July and October 2015, which provided our matchup dataset. The two inset maps show the location of each plume on Sumatra (first map) and Kalimantan (second map), with peatland areas shaded according to Global Forest Watch (http://data.globalforestwatch.org/datasets/).

As introduced in [38] and presented in Section 2, in order to establish an appropriate TPM $C_{e}$ for a particular biome, collocated satellite-derived observations of smoke plume AOD and FRE are required for a set of matchup fires statistically representative of the biome’s fire events. To derive $C_{e}$ for the type of highly radiant peatland fires we study here, we found it necessary to further modify parts of the original methodology to address three specific issues:

(i) In [38], entire plumes were manually digitised from the satellite imagery to create the southern African fire matchups. The radiant heat output (FRP) of the largest fires investigated in SE Asia is more than an order of magnitude higher than those in that original study, however, and their extensive smoke plumes often merge and/or have indistinct boundaries—making accurate delineation of a fires entire plume often impossible. There is also far more significant potential for cloud contamination of the plume observations in the SE Asian environment (see Figure 1 and Figure 2).
(ii) In [38], it was assumed that each plume analysed had been produced between the start of the most recent diurnal cycle of the associated fire and the time of the polar orbiting satellite overpass used to generate the AOD product. However, certain of the SE Asian fires did not show obvious FRP minima during the night, meaning that the start time of the temporal integration period over which FRE was calculated could not be determined in the same way.
(iii) The extreme optical thickness of the peatland fire plumes means parts of them are often incorrectly masked as meteorological cloud by satellite AOD products, or given an unrealistically constant maximum AOD (this includes the standard MODIS AOD products employed by [38]), potentially resulting in low biased $C_{e}$ estimates.

To deal with issue (i) and select an appropriate matchup between the TPM in a given plume and the FRE for the fire that produced it, only a spatial subset of each plume was digitised (as shown in Figure 2), rather than the entire plume. This avoided the need to identify the complete plume extent, greatly reduced issues of cloud contamination, and enabled us to exclude areas of the plume that had merged with smoke from other fires. In parallel with these advantages, only using part of the plume also resolved issue (ii), since we could then integrate the FRP values only over the time that the part of the plume used for the TPM calculation had been generated. Identifying this time period then became one of the most complex steps in the calculation, and the approach used to do so is discussed in Section 3.2. To resolve issue (iii), a modified AOD retrieval approach, able to provide AOD estimates for very optically thick smoke, was developed, as discussed in Section 3.3.

Using this modified approach, $C_{e}$ was then generated for the highly radiant SE Asian peatland fires using a suitable set of potential target plumes identified using the NASA Worldview webtool (https://worldview.earthdata.nasa.gov). MODIS and VIIRS true colour composite browse imagery were comprehensively searched over the islands of Kalimantan and Sumatra from July through October 2015, and ultimately thirteen plumes (from tens of potential plumes) were identified that contained digitizable subsets (for detailed plume information see Table 1 contained in Section 4.1). These subsets were considered of sufficient length for reliable calculation of the time taken for the plume to be produced (see Section 3.2, typically tens of km), had sufficient high quality AOD retrievals for reliable estimation of TPM in optically thick conditions (see Section 3.3), and had collocated FRP observations coming from Himawari-8 AHI active fire pixels with each fire confirmed as being located on peatland (see Figure 2) using the SE Asian peatland shape files available from Global Forest Watch (http://data.globalforestwatch.org/datasets/). All data for the 13 digitized plumes were reprojected to a UTM grid with a 750 m pixel resolution, chosen to reflect the nominal resolution of VIIRS M-band pixels (see Section 3.3) at or close to nadir.

### 3.2. Temporal Integration of FRP to FRE Using Plume Velocity Estimates

To estimate each fire’s FRE, we used the FRP measures for each of the active fire pixels detected within the digitised fire and plume polygon, as discussed in Section 3.1. These FRP measures, expressed in watts ×10^6^ (MW), were calculated from level-1b Himawari imagery [26] at the locations of active fire pixel detections made using an adaption of the geostationary fire thermal anomaly (FTA) algorithm prototyped in [46] and described in detail in [30]. The FRP measures themselves were calculated using the MIR-radiance method of [16,47]. The FRE (in joules ×10^6^; MJ) for each fire was then calculated from the temporal integration of its FRP measures. This was easily achieved due to the 10-min imaging frequency of the Himawari-8 data that avoided the need for interpolation during long gaps between FRP observations. The time interval for each FRP temporal integration was set to be that over which the matching plume subset (Figure 2) was produced. This was calculated from the plume subset length (m) and horizontal velocity (m·s^−1^). The former was determined by creating a vector p from the plume’s approximate distal end (identified as the approximate midpoint of the distal end during digitisation) to the mean UTM-projected location of the Himawari-8 active fire pixels contained within the plume polygon. The total length of the plume subset is then defined as ‖p‖. Plume velocity was derived from consecutive Himawari-8 0.55-µm images using the OpenCV Farneback optical flow algorithm [48] (see Appendix B) applied to the seven AHI images preceding each of the VIIRS overpasses used to provide the plume AOD measure (see Section 3.3). Seven images, when paired, provided a 1-h subset of Himawari-8 observations from which to estimate the flow. This was deemed suitable as most plume subsets, when evaluating their flows, were produced by the associated fire in less than an hour. From the image flow estimates, plume velocity, w (m·s^−1^) is estimated as:

(1) $$w=\frac{\underset{i}{\max}\left\|({\bar{x}}_{flow,\;i},{\bar{y}}_{flow,\;i})\right\|}{s}$$

where i defines the set of geostationary image pairs between which the flow is calculated, ${\bar{x}}_{flow,\;i}$ is the mean subpixel shift (i.e., flow) in the UTM projected geostationary image *x*-axis with the mean calculated only from pixels contained within the plume subset polygon, and ${\bar{y}}_{flow,\;i}$ is the same but for the *y*-axis. The denominator s is the total number of seconds between temporally adjacent Himawari image acquisitions, which occur every 10 min (i.e., 600 s).

**Figure A2:**
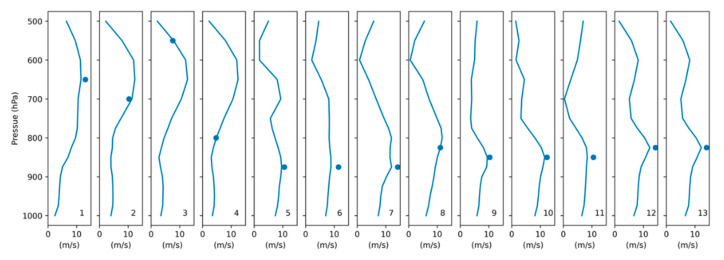
Comparisons of spatially and temporally collocated ERA5 wind-speed magnitudes (calculated from u and v components) against in-plume horizontal velocity retrievals derived from the Farneback optical flow algorithm. The in-plume horizontal velocity is matched to the closest ERA5 velocity across pressure levels, with most plumes injecting near 850 hPa (~1.5 km above mean sea level), the height value assumed in [44].

The flow measures, ${\bar{x}}_{flow,\;i}$ and ${\bar{y}}_{flow,\;i}$, are subject to two screening steps prior to calculating their mean: (i) to exclude any flow pixels not physically representative of the plume-indicated wind direction, the angle between all flow vectors and p is calculated, and only flow vectors within $\pm \frac{\pi }{8}$ radians of the plume vector are retained; (ii) some nonplume flow pixels are sometime contained within the plume polygon, typically providing flow estimates for the land surface (i.e., 0) and are excluded. Finally, the image pair with the maximum of the screened flows is retained as it was found to be the most reliable indicator of plume speed based on comparisons to ECMWF Re-Analysis 5 (ERA5) wind speeds (see Appendix B, Figure A2).

Total time for the plume subset to be produced was calculated as $t=\|p\|\cdot w^{-1}$. It is this duration *t* that was used as the time period over which the matching FRP time-series for the fire was integrated to estimate FRE. The end of the integration period was the time of the VIIRS overpass providing the AOD estimate, whilst the start of the integration period was *t* seconds earlier.

### 3.3. TPM Estimation

AOD retrievals for each plume subset (Figure 2) were derived from the VIIRS multispectral 750-m spatial resolution “M-band” observations via two approaches. The first for pixels showing optically thick conditions (i.e., extreme AODs, here >2.0) and the second for all other pixels. For the latter, AOD values from the standard VIIRS aerosol product were used, based on land pixels whose 555 nm AOD was derived using a modified dark target (DT) algorithm (originally developed for MODIS) [49]. The modified algorithm uses five M-bands (M1: 0.412 µm; M2: 0.445 µm; M3: 0.488 µm; M5: 0.672 µm; M11: 2.25 µm) to estimate AOD, and a key enhancement over its use with MODIS is that five aerosol types are evaluated in the retrieval’s optimisation step (compared to the two assessed in the MODIS DT product), and these include both low- and high-absorption smoke aerosols taken from AERONET inversion climatologies [50]. The VIIRS AOD product is available from the NOAA CLASS data centre as an aggregated environmental data record (EDR) at a nominal 6-km spatial resolution, or an unaggregated pixel-level intermediate product (IP). The IP product was used herein because its higher spatial resolution better captures the AOD variability within each plume. Retrieval confidence flags are produced at the IP level and used in EDR generation, ranging from 1 (good) through 4 (non-retrieved), and AODs higher than 2.0 are considered invalid, even though they are common in biomass burning plumes [51].

For pixels showing extreme AODs, no publicly available satellite-derived standard AOD product currently exists that retrieves AODs greater than 5, even though such values exist in the SE Asian biomass burning plumes of 2015 [51]. Therefore, to provide data for these pixels we deployed the Oxford-RAL Aerosol and Cloud retrieval algorithm (ORAC) for all pixels showing optically thick conditions (AOD > 2.0). ORAC is able to retrieve AOD even in such extreme AOD situations [52], but was not used for the optically thin (AOD < 2.0) condition since when applied to data from single-view sensors such as VIIRS (as opposed to dual-view sensors such as ATSR), the retrievals retain significant and unwanted sensitivity to changes in the underlying land surface reflectance [52]. We applied ORAC to data from VIIRS channels M3 (0.488 µm), M4 (0.555 µm), M5 (0.673 µm), M7 (0.865 µm), M8 (1.24 µm), and M11 (2.25 µm), parameterising the retrievals with the smoke optical properties reported in [50] for tropical forest fires in the Amazon. These are unlikely to be a perfect match for the smoke coming from SE Asia fires, which, for example, will have very likely been contributed to by organic soil burning, so wherever possible we compared the ORAC-retrieved AODs from SE Asian plumes to those derived from the surface AERONET network (which itself provides data suitable for retrieving AOD in even optically thick conditions). A bias was noted between the ORAC and AERONET AOD retrievals made in the extreme AOD conditions considered here, believed most likely to arise from differences between the assumed and true optical properties of the smoke particulates. This bias was easily adjusted for by using a single linear scaling factor (see Appendix A).

Even with the two AOD retrieval methods noted above, complete plume subset coverage was not always achieved, and quality indicators were used to screen out low quality AOD retrievals. Specifically, only VIIRS IP data with quality of 2 or better and ORAC retrievals with costs of ≤10 (a limit applied previously [53]) were used, with the ORAC cost providing an indication of the consistency between the retrieved and observed atmospheric state [52]. The AODs of any non-retrieved pixels were estimated using a radial basis function interpolation approach. This was found to provide the most representative AOD estimates when evaluated against alternative interpolation approaches on artificially removed AOD pixels (see Appendix C).

Excess AOD was calculated for every pixel in each plume subset by differencing the pixel’s AOD value from that derived for the upwind plume background (defined from an additional polygon drawn immediately upwind of the fire during the digitisation process). The total particulate matter (TPM, g) in the plume subset was then calculated from the excess AOD totalled over all pixels using:

(2) $$TPM=A\cdot AOD_{e}\cdot \beta^{-1}$$

where A is the area (m^2^) of the plume subset polygon, $AOD_{e}$ (unitless) is the summed excess AOD for the plume subset, and β is the mass attenuation coefficient (m^2^·g^−1^) for the constituent smoke.

The selection of an appropriate mass attenuation coefficient depends on numerous and largely unknown factors associated with a fires environment, and the usual solution is to assume a globally or regionally appropriate value, such as the 4.6 m^2^·g^−1^ applied in [45] or the regional mean of 3.5 ± 1.0 m^2^·g^−1^ used for Southern African fires in [38]. However, peatland fires generally involve organic soil combustion as well as combustion of vegetation, and thus a different mass extinction coefficient may be warranted compared to smoke coming from a purely surficial vegetation fire [54]. However, since the fires we focus upon are highly radiant and clearly involve substantial flaming combustion of vegetation (Section 4.2 and Figures 1, 5, and 6), we retain use of the most widely applied biomass burning smoke mass extinction coefficient of 4.6 m^2^·g^−1^ [44]. Further investigations of this parameter based on field or laboratory measures is suggested as a future research priority. Using this smoke mass extinction coefficient allowed us to generate TPM values for the plume subsets of our matchup fires, and combining these with the per-fire FRE values we generated the smoke emissions coefficient *C_e_* using ordinary least squares (OLS) regression forced through the origin.

## 4. Results and Discussion

### 4.1. Derivation of TPM Emission Coefficient (C_e_)

**Figure 3:**
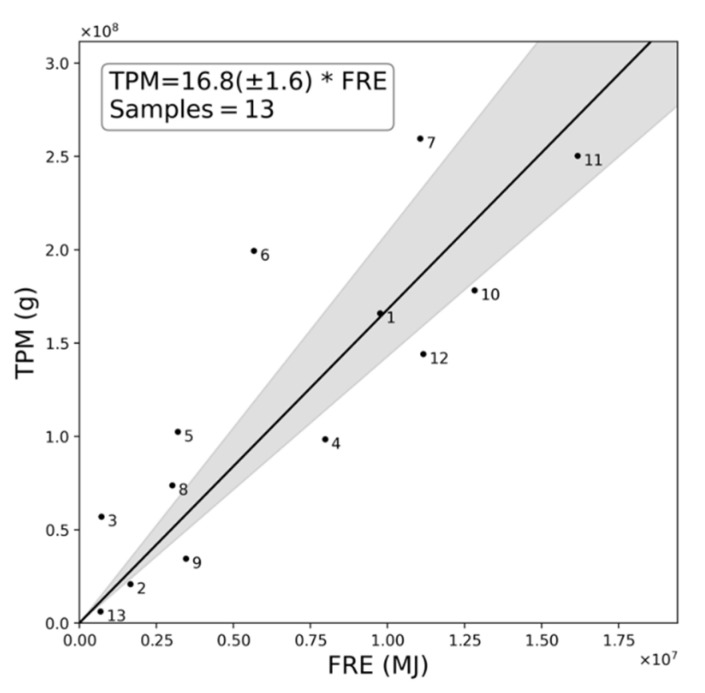
Comparison of total particulate matter (TPM) in the plume subset and matching fire radiative energy (FRE) released from the causal fire, shown for the thirteen matchup fires of Figure 2, with each numbered as shown therein. The ordinary least squares (OLS) linear best fit through the origin is used to generate a TPM smoke emissions coefficient (*C_e_*; 16.8 g·MJ^−1^) appropriate for this type of highly radiant SE Asian peatland fire, with the grey shaded area indicating the 95% confidence interval [14.3 g·MJ^−1^, 20.9 g·MJ^−1^].

Figure 3 shows the data from the thirteen matchup fires of Figure 2, used to estimate *C_e_* as 16.8 ± 1.6 g·MJ^−1^ through OLS regression, with Table 1 reporting various metrics for each matchup. For comparison, in Table 2 are the existing TPM $C_{e}$ published for SE Asian peatland fires, either derived from the multiplication of an FRE to fuel consumption conversion factor with a TPM emission factor [27], or calculated directly via a “top-down” method relating FRE to TPM emissions [37]. Values range from 69.3 g·MJ^−1^ in the former case to 52.4 g·MJ^−1^ in the latter, and are far higher than the 16.8 ± 1.6 g·MJ^−1^ determined herein. The highest *C_e_* (69.3 g·MJ^−1^) was derived using a largely “bottom-up” approach where a dry matter fuel consumption rate conversion factor of 5.87 kg·MJ^−1^ was established though comparison of GFAS-derived FRP values against burned-area-derived peatland fuel consumption estimates coming from GFED [27], and the 11.8 g·kg^−1^ TPM emissions factor from deforestation fires [33]. A recent update to the EF from [32] provides an explicit, though indirectly estimated, TPM EF value for peat of 27.5 g·kg^−1^, resulting in an even larger *C_e_* estimate of 161 g·MJ^−1^. The “top-down” *C_e_* of 52.4 g·MJ^−1^ was calculated from a set of 19 collocated smoke plumes and fires burning on SE Asian peatlands, with the TPM measurements derived from MODIS deep blue AOD products [55] and the FRE calculated from MODIS FRP data provided by successive overpasses of the Terra and Aqua satellites and an assumed FRE diurnal distribution [37].

### 4.2. Discussion of TPM Emission Coefficient (C_e_) Differences

The TPM $C_{e}$ reported in [27,37] for SE Asian peatland fires are very substantially larger than the 16.8 g·MJ^−1^ value generated herein (Figure 3). A key cause of this difference is suggested to be the fact that unlike our highly radiant peatland fires, which contain substantial areas of flaming combustion (Figure 1), many fires on peatlands tend to be solely smouldering, which generates far more particulate matter emissions per unit of dry matter burned than do flaming fires [21].

**Figure 4:**
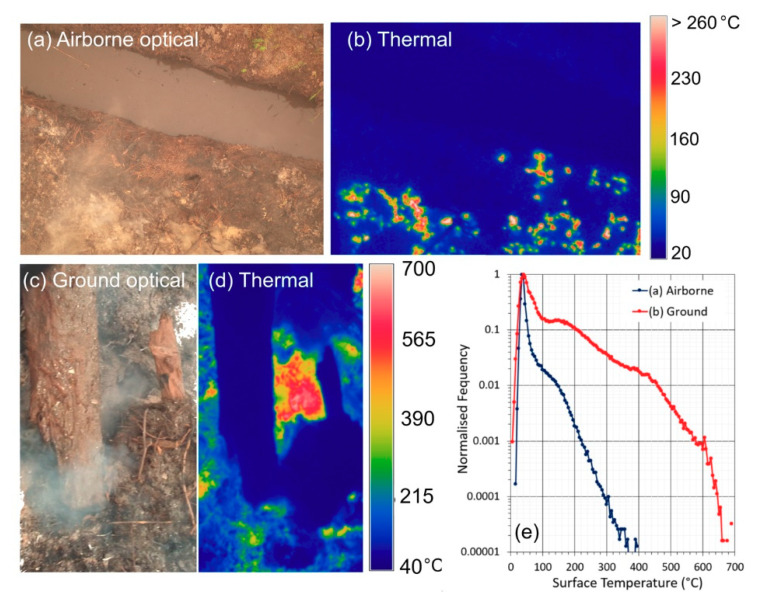
SE Asian peatland fire infrared surface temperatures and matched optical imagery. Data come from infrared camera measurements conducted on 14 October 2015 during the Central Kalimantan field campaign described in [10], and were acquired close to location 3 detailed therein. (**a**) Example airborne optical image of subsurface peat burning in a largely treeless area next to a drainage canal, captured from a UAV flying ~20 m height above ground, along with (**b**) the matching thermal (8–14 µm) brightness temperature image. (**c**) Example ground-based optical image of peat burns in a more forested area, where hot smoke is escaping from vents and holes and there is more woody material to burn. (**d**) Matching thermal brightness temperature image. (**e**) Normalised frequency of surface temperatures collected in the two different areas from multiple examples of the type of airborne and ground-based thermal imagery shown in (**b**,**d**), and where the more forested locations (**c**,**d**) show significantly higher temperatures, albeit still primarily coming from subsurface combustion.

A second cause is related to the typical surface temperatures found at locations of burning peat, which are typically far cooler than those of surface vegetation fires. In [16], it is shown how the MIR-radiance method of FRP retrieval is adapted to a temperature range relevant to smouldering and flaming-phase vegetation combustion. Whilst the approach greatly benefits from not requiring knowledge of the actual fire effective temperature, it is tuned for temperatures exceeding 650 K (considered a good lower limit for smouldering phase activity [56]). Below this temperature, the radiative power output becomes increasingly underestimated unless the FRP coefficient is rederived for a cooler temperature range [16]. Such FRP underestimation at lower temperatures is in some cases advantageous, because in standard vegetation fires such temperatures are mostly associated with recently burned but still cooling ground that is not consuming fuel. However, by burning underground, subsurface peat fires can still consume fuel whilst generating surface temperatures far lower than 650 K [57], meaning the equivalent FRP to fuel consumption rate conversion factor would need to be increased compared to that for standard surface smouldering or flaming fires. This may be one reason that during comparisons between MODIS-derived FRP measures and GFED-derived fuel consumptions, [27] found an “FRE-to-fuel consumption” conversion factor for savanna fires rather similar to that of the small-scale laboratory vegetation burns of [16], but for peatland fires the estimate was more than an order of magnitude higher. To demonstrate, Figure 4 shows infrared surface temperature data of SE Asian peatland fires acquired by us during the October 2015 Kalimantan fieldwork described in [10]. The data come from two areas of burning peat. The first area is a largely cleared location next to a drainage canal imaged from a low altitude UAV (Figure 4a,b). Ambient surface temperatures are below 313 K (40 °C) and elevated temperatures at the areas of combustion are mostly lower than the aforementioned 650 K (377 °C) threshold used by default in the MIR-radiance method. These elevated surface temperatures appear mostly to be generated via heat conduction from below at locations where smouldering peat consumption is proceeding subsurface, often below a layer of white ash. The second area is a more forested location (Figure 4c,d), where the thermal imagery was acquired from the ground. Here, some surface woody material is being consumed by smouldering combustion, but the vast majority of elevated surface temperatures, and certainly the hottest ones, are from areas of peat and appear to be actively generated by convective heat transfer related to the passage of hot smoke through vents and cracks in the peat surface. Substantial areas exceed the 650 K (377 °C) threshold, though most are still cooler than this—as confirmed by the normalised frequency distribution shown in Figure 4e.

A third cause for the difference between the $C_{e}$ estimate determined herein and the far higher values determined by [27,37] is that when examining wide-scale peat fires with moderate to low spatial resolution spaceborne data, the pixels successfully triggering the active fire (AF) detection algorithm typically comprise many individual subsurface fires, which together can present a sufficiently significant surface thermal signature to be detected. However, there will likely be many fire regions with insufficient high temperature areas to trigger the AF detection algorithm, meaning their FRP will not contribute to the FRP total, but may well contribute significantly to smoke production or estimated BA-derived fuel consumptions. Such FRP underestimation caused by undetected AF pixels will likely be greatest in the case of subsurface smouldering peat fires, where far more of the pixel area must be covered by the elevated temperature material for the AF detection algorithm to identify the pixel as containing a fire, compared to fires at flaming temperatures.

**Figure 5:**
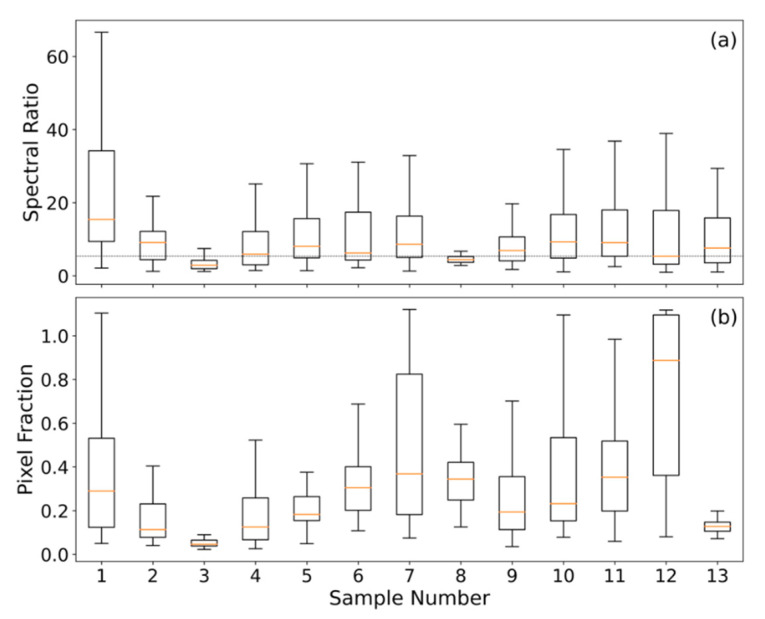
Results of the investigation as to the nature of the thirteen matchup peatland fires focused on herein and shown in Figure 2. (**a**) Boxplots of the “excess above background” 3.9 to 11 µm spectral radiance ratio for the Himawari active fire pixels making up each fire, along with that associated with a 700 K fire (5.4 W m^2^ sr^−1^ µm^−1^ (W m^2^ sr^−1^ µm^−1^)^−1^); shown by the dashed vertical line. (**b**) Boxplots representing the fraction of each Himawari pixel that would be required to be covered by a subsurface smouldering peat fire having an effective surface temperature of 412 K (mean of the surface temperatures determined in [57]). Himawari-8 active fire pixels are those detected within the associated plume polygons shown in Figure 2 in the hour prior to the VIIRS afternoon overpass. In both (**a**) and (**b**), the boxplots follow standard conventions showing the median (red horizontal lines), interquartile range (box), and minimum and maximum values (whiskers).

**Figure 6:**
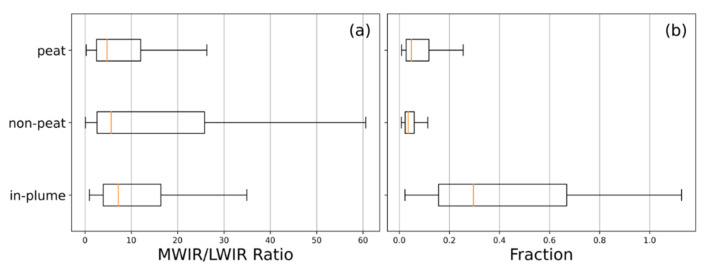
Spectral ratio (**a**) and subpixel active fire fraction (**b**) for AHI-detected active fire pixels contained in our matchup fires against all AHI-detected active fire pixels for September and October 2015 identified in the study region (latitude range: 10° N:10° S; longitude range: 100° E:120° E). Active fire pixels are separated into those burning on peatlands and on non-peatlands using the Global Forest Watch peat map shown in Figure 2. As with Figure 5, the boxplots in (**a**,**b**) follow standard conventions.

**Figure A4:**
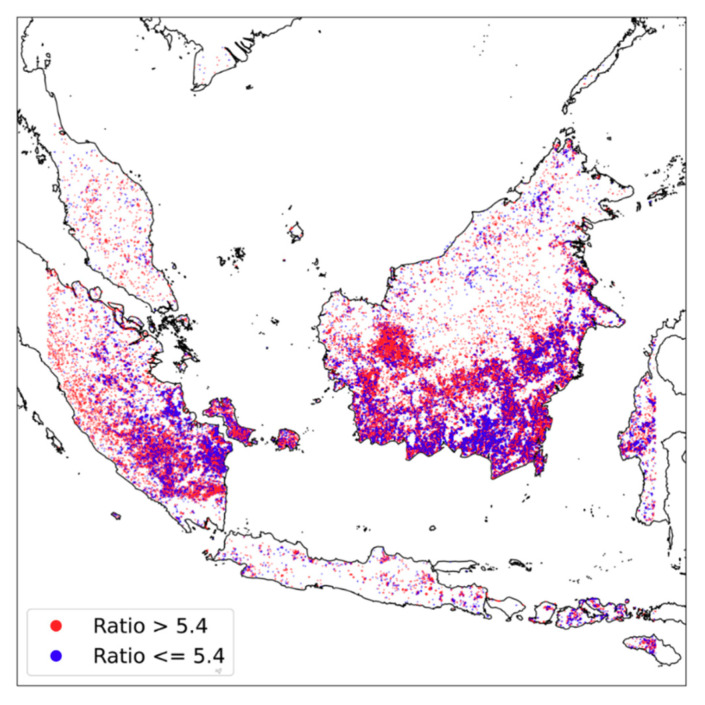
Map of the study region showing gridded (1-arcminute; ~2 km) AHI active fire detection locations for September and October 2015, coloured by the median spectral ratio for each grid cell (defined in Section 4.2).

**Figure A5:**
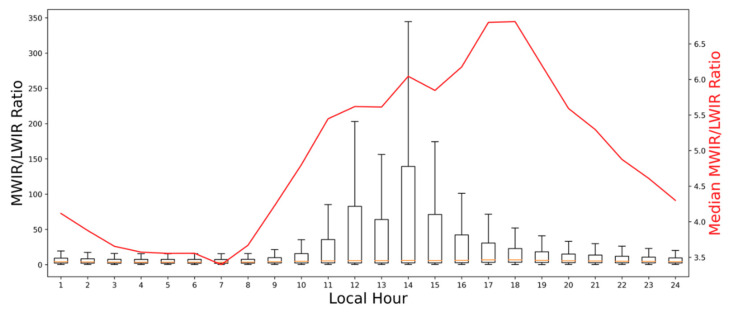
Spectral ratio (defined in Section 4.2) boxplots for September and October 2015 for the region shown in Figure A4, aggregated into hourly bins (local solar time). Red line (RHS, *y*-axis) provides a zoomed view of the median value for each boxplot. The boxplots follow the same conventions described in the Figure 5.

The above factors provide an indication as to why the *C_e_* values established previously for peat fires, assumed to comprise a significant subsurface smouldering combustion component, are so much higher than those established for the potentially more flaming-dominated fires assessed herein. Confirmation of these fires’ flaming nature comes from several sources. Firstly, exploiting the large temperature differential between flaming and smouldering combustion in SE Asian peatland fires (~400 K, [57]) allows the spectral ratio method demonstrated in [58,59] to discriminate between them. This was conducted using the ratio of the excess (i.e., above background) mid- and long-wave infrared (MWIR and LWIR, respectively) spectral radiances available in the AHI FRP product at the location of detected active fire pixels [26]. Comparing this against an empirically defined threshold value of 5.4 (W m^2^ sr^−1^ µm^−1^ (W m^2^ sr^−1^ µm^−1^)^−1^), equivalent to a combustion temperature of 700 K, allowed us to identify whether each AF pixel was dominated by areas of combustion cooler or hotter than this (validation of the defined threshold against known flaming locations is provided in Appendix E). Assessing AHI-detected fires burning across the SE Asian study region on a 1-arcminute grid aggregated over the months of September and October 2015 (see Appendix D, Figure A4), showed that approximately ~51% of the peatland grid cells have a median spectral ratio of >5.4 (i.e., hotter than 700 K) compared to ~65% of grid cells not located on peatlands, a statistically significant difference (*p* < 0.01) in terms of temperature. Aggregating the data from peatlands into hourly bins (see Appendix D, Figure A5) returns median spectral ratio values of >5.4 from 12:00 to 20:00 local time, an indication that flaming combustion is more prevalent during the day, as might be expected since daytime conditions tend to promote more intense fire activity [9,24]. These findings together indicate that this spectral ratio thresholding is suitable for discriminating between more flaming and more smouldering dominated areas of combustion, and that smouldering combustion is more prevalent in peatland areas and at night. Figure 1 (inset) also proves the presence of substantial areas of flaming combustion, as do the spectral ratio values for the 13 peatland fires of Figure 2, which are shown in Figure 5a, with the median spectral ratio of all but two of the fires exceeding the defined threshold of 5.4. A further comparison of the spectral ratio values for the AHI active fire pixels of our matchup fires was made against all active fire pixels detected in peatland and non-peatland areas in the study region across September and October 2015 (Figure 6a). The median spectral ratio for the matchup fires (7.2) is significantly higher than that for the wider peatland (4.8) and non-peatland (5.7) areas, indicating that the matchup fires have substantially more flaming combustion. Note that the median spectral ratio across all peatland fires is less than the threshold of 5.4, indicating the predominance of smouldering combustion in peatland areas (but not in our highly radiant matchup fires).

A second assessment is made in Figure 5b, where for active fire pixels associated with each matchup fire we estimate the required subpixel fraction *f* that would need to be combusting to generate the observed FRP, assuming a 412 K (139 °C) surface temperature (the mean smouldering peat fire temperature determined in [57]):

(3) $$f=\frac{FRP\cdot {\left(\frac{\sigma }{a}\cdot \beta \left(\lambda ,T\right)\right)}^{-1}}{A_{s}}$$

where f is the fractional area (unitless); FRP is the fire radiative power (W), $\frac{\sigma }{a}$ is the AHI specific FRP coefficient (sr µm); β(λ, T) is the Planck function used to define spectral radiance (W m^2^ sr^−1^ µm^−1^) at wavelength λ (µm), here 3.9 µm and temperature T (K), here 412 K; and $A_{s}$ is the area (m^2^) of the AHI pixel (4 km^2^ at the subsatellite point). Given the relatively large AHI pixel areas, the resulting fractional areas coming from application of (3) to our matchup fires appear unrealistically high, with median pixel fractions of >20% for the majority of these fires. This contrasts against the same estimate of subpixel fraction calculated for all active fire pixels detected on peatland and non-peatland areas (Figure 6b), which indicates a far more reasonable median pixel fraction of ~0.05% (peatland) and ~0.04% (non-peatland). This difference suggests (i) that our matchup fires must contain substantial areas of combustion far hotter than 412 K (which would then make their subpixel fractional areas far lower and more realistic), and (ii) that they are among the most radiant fires occurring during the 2015 El Niño period in the study region.

Together, the data from these three assessments support the interpretation that our matchup fires are far more radiant and contain far more flaming activity than do the majority of the peatland fires identified by AHI during September–October 2015, and since more than 50% of them are located on Indonesian governmental peatland concessions for wood fibre (6 samples) or palm oil (1 sample) plantations (see Table 1), substantial surface vegetation and vegetation litter is indeed likely to be available at these sites to support flaming combustion of the type shown in Figure 1. Thus, it follows that the *C_e_* established for our targeted highly radiant fires is associated with peatland burns containing a significant flaming phase component.

### 4.3. Consideration of Contributions to Uncertainties

As is apparent in Figure 1, wildfire plumes are rather transparent in the SWIR and MWIR spectral regions, because these wavelengths are significantly longer than typical smoke particle diameters. However, the extreme optical thickness of the plumes of our matchup fires likely does provide some reduction on the MWIR radiance measures used to establish FRP [60], though the magnitude and variability of this is currently uncertain and the FRP values are thus only atmospherically corrected for the effect of atmospheric water vapour and trace gases [26]. This, along with active fire detection errors of omission, results in an increased *C_e_* since the measured FRP is low biased. As discussed in Section 4.2, *C_e_* values derived using AHI might be expected to be lower than those produced using MODIS (assuming AOD values were correctly specified in both cases), given the reduced capability of AHI to detect less radiant fires due to its coarser nadir point pixel size [26,46] (up to 66% active fire detection omission rate [26]). Such active fire errors of omission are, however, to some extent accounted for in our approach—since whilst some active fire pixels in our matchup fires that are burning below the AHI minimum FRP detection threshold (~40 MW [26]) may remain undetected, their TPM contribution is captured in the plume and incorporated into the derived *C_e_*. Thus, as long as the *C_e_* derived herein is applied to AHI data and to fires with characteristics similar to those of the matchups focused on here, errors will be minimised. This does, however, highlight the need for an approach to effectively determine whether a fire is combusting predominantly in the flaming or smouldering phase, since our derived *C_e_* has been focused on the more radiant peatland fires that contain substantial flaming activity. The spectral ratio method applied herein demonstrates one potentially suitable approach for doing so.

Further uncertainties are introduced during the establishment of the temporal integration period under the situation where smoke plumes are constantly evolving, with significant changes in their appearance, sometimes occurring between AHI image acquisitions, which make tracking their motion challenging, especially for some of the limited plume extents used herein. Such problems are minimised by the very high 10-min temporal resolution of AHI, which allows a smaller window size to be used to capture image motion, and by the strict postprocessing applied in the extraction of reliable motion estimates. Furthermore, comparisons made against ERA5 profiles of wind speed, shown in Appendix B, demonstrate that the horizontal wind speed estimates produced using our motion tracking approach to the AHI-detected plumes are reasonable, with differences of <2 m·s^−1^ in all instances.

The AOD estimates used to generate the TPM measure of our matchup fires have four main sources of identified uncertainty: the interpolation of missing AOD values; the assumed ambient background AOD; the issues of estimating AOD in conditions of optically thick smoke; and inconsistent wind speed over the plume cross section, such that AODs observed within the plume are not fully resultant from the fire during the FRP integration time period. Estimating appropriate AODs for missing pixels is perhaps the most challenging problem of the four. In [37], this issue was tackled using the maximum near-fire-observed AOD value, but since the cause of the missing data is often related to the fact that a pixel’s AOD exceeds the maximum possible with the retrieval approach, or that the smoke is optically thick and misidentified as cloud [51], assigning the maximum “unsaturated” AOD value found elsewhere in the plume will certainly lead to a low biased AOD estimate. The consequence would be a low biased in-plume TPM estimate and resulting *C_e_* coefficient. By contrast, the radial basis function interpolation method deployed herein is able to provide interpolated AOD estimates even exceeding the maximum observed at retrieved pixels, by providing an estimate influenced by the gradients of the proximal AOD values. Various approaches can be employed to generate background AOD estimates, [38] for example, use of a regional value, but this seems inappropriate for conditions studied herein where very significant spatial variations in ambient aerosol load are seen over small spatial scales. We assumed that the AOD measured immediately upwind of the plume (as determined by the plume velocity direction) is that which is representative of the background AOD, since it should not be contributed to by the fire’s smoke emission. In [51], the potential for accurately retrieving AOD > 5 optical depths is shown to be problematic, but even if similar limitations apply to the optically thick AOD retrievals conducted with the ORAC retrieval approach used herein, only an average of 6% of pixels across the 13 plumes shown in Figure 2 have optical depths above 5 (a maximum of 15.4% of pixels in plume 11), and so the overall effect will be limited. The effect of differing wind speed across the plume cross section is difficult to ascertain, and has not been considered in previous work where temporal plume subsetting methods similar to those employed here are used [44,45]. For plumes that are not injected above the boundary layer, the inclusion of aerosol material from outside the FRP integration period in the TPM calculation is more likely due to increased turbulence [61]. For plumes that are injected above the boundary layer, there is less turbulence [61] and so it may be more reasonable to assume that the AOD contained within a plume cross section is more closely associated with an instant of the producing fire. Given that most of the fires evaluated here are very large and have well-defined plumes, above boundary layer injection seems probable, and we therefore assume that the TPM contained in the plume is well correlated with the FRE, as indicated by Figure 3.

### 4.4. Significance of Flaming Phase Dominated Fires

**Figure 7:**
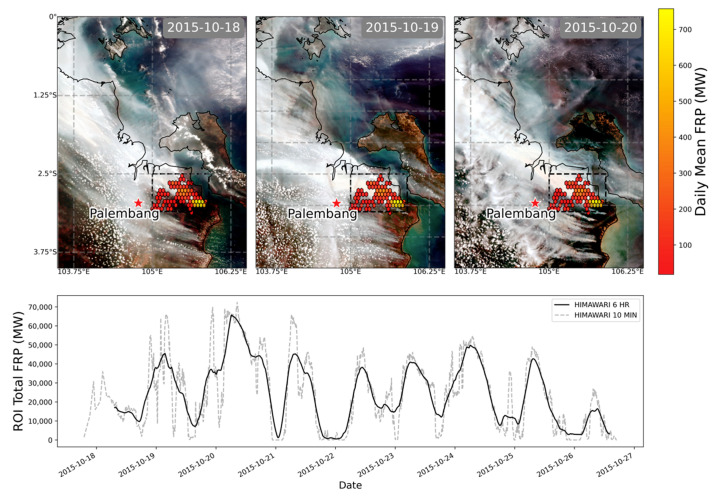
Data of the highly radiant Sumatran peatland fire associated with Plume 12 of Figure 2, and confirmed as showing flaming as well as smouldering activity in Figure 5. The upper plot shows the smoke plume and the spatial distribution of mean Himawari-8 derived FRP for three days of the fire. The lower plot shows the nine-day time series (each tick represents a 24-h period) of total Himawari-derived FRP for the fire (contained in the black bounding box marked in the images), with the dashed line showing the original 10-min resolution data and the solid line a 6-h sliding window. The data from this fire used in the matchup process to derive *C_e_* (Figure 2 and Figure 3) came from the 20 October 2015 record (Table 1).

Since the SE Asian peatland fire regime is often assumed to be dominated by persistent smouldering combustion [18,62], it is worthwhile to consider the significance of the highly radiant flaming-phase peatland fires that comprise our matchup dataset and how they contribute to the overall smoke emission totals of the region. To do this, we studied the signatures of the most radiant matchup fire in our dataset, that associated with Plume 12 in Figure 2. This Sumatran fire burned from 18 to 27 October 2015 according to our AHI-derived FRP time series (Figure 7), and released 1.76 × 10^10^ MJ of fire radiative energy. Applying our “top-down” *C_e_* to this FRE total results in a total emitted particulate matter mass of ~0.3 Tg for this fire alone, which represents around 9% of particulate emissions from all Sumatran fires burning in September and October 2015 (assuming ~3 Tg of PM_2.5_ released during this period and that PM_2.5_ typically comprises 90% or more of emitted PM mass totals [10,21], giving ~3.4 Tg of total particulates). Thus, this single fire alone markedly contributes to regional TPM emission totals, and our matchup dataset shows thirteen such intensely burning highly radiant peatland fires, whose TPM emission rates are each far higher than those of the far more common low intensity smouldering peat fires. Combined, the TPM emissions of the twelve other fires studied totals to ~0.4 Tg of particulate matter mass. To demonstrate the inappropriateness of applying existing *C_e_* values (Table 2) to these types of high intensity peatland fire, along with the updated “bottom-up” value of 161 g·MJ^−1^ derived from [32], we calculated the equivalent TPM total for the fire associated with Plume 12. TPM totals of 0.93, 1.22, and 2.84 Tg were calculated for this single fire from the alternative coefficients, which are clearly too high given the total from all Sumatran fires is estimated to be ~3.4 Tg [10].

The high Himawari-derived FRP values of the matchup fires shown in Figure 2 mean they are consuming significant amounts of fuel per unit time. This is probably occurring in spreading flaming fire fronts of the type shown in Figure 1, and in fuels consisting of both surface vegetation as well as organic peat soil. In [10], it was also found that the particulate matter emissions factor for this type of flaming vegetation fire atop burning peat is significantly higher than for smouldering peat fires alone, further increasing the amount of particulate matter they release per unit time. Overall, our results demonstrate that indiscriminate application of a single *C_e_*, for example, from [27] or [37], based solely on the fact that the landcover type is peatland is inadvisable.

## 5. Conclusions

Our analysis focused on a series of the most radiant fires burning in Indonesian peatlands during the 2015 El Niño (September and October 2015). These fires were responsible for the highest fuel consumption rates and thus the highest rates of PM emission during this event. We generate a matchup dataset of these fires and use it to generate a TPM *C_e_* estimate of 16.8 ± 1.6 g·MJ^−1^, which can then be applied to all FRP data of this type of fire to estimate TPM emissions per unit time (g·s^−1^), even in real-time. Our derived *C_e_* is far lower than those currently published for SE Asian peatland fires, which typically exceed 50 g·MJ^−1^ [27,63]. The difference is primarily attributed to the fact that the highly radiant fires focused upon herein include significant flaming activity in surface fuels, such as forest and plantation vegetation, as well as smouldering combustion within the peat. Deriving a single *C_e_* value for all SE Asian peatland fires, which area-wise tend to be dominated by subsurface smouldering combustion, is the likely reason for the far higher smoke emissions coefficients coming from past studies. Our results have important consequences for the estimation of particulate matter emissions for SE Asian peatland fires using the FRP approach, since we show that application of an inappropriate *C_e_* derived for smouldering fires can result in highly biased PM emission estimates when applied to the more intensely radiant fires that can also be found on SE Asian peatlands. Indeed, these latter types of fire can be highly important to overall PM emissions totals and are thus important to consider accurately. We estimate that the single most radiant Sumatran fire assessed herein produced approximately 9% of all PM_2.5_ emissions originating from Sumatran fires in September and October 2015. Such large fires can greatly affect regional air quality, including in highly populated parts of Indonesia and neighbouring countries such as Singapore [24,25].

Our work demonstrates for the first time the importance of considering combustion phase both when establishing *C_e_* for a particular biome in which very different types of fire (smouldering dominated vs. mixed phase or flaming dominated) can occur, and during application of the resulting coefficient to subsequent FRP observations. The FREM approach and our optimised *C_e_* coefficient appear well suited for use in fire emissions inventorying and air quality early-warning, and indeed the highly radiant, surficial flaming fires we focus on herein are more energetic and thus more likely to inject pollutants into the free atmosphere that may assist their transboundary transport [64]. Further work is recommended to determine the most appropriate smoke mass extinction coefficient for peatland fires and for best discriminating areas of flaming- and smouldering-dominated combustion to ensure the most appropriate *C_e_* is applied to each type of fire.

## Figures and Tables

**Table 1:** Key statistics for the thirteen matchup fires shown in Figure 2 and used to derive the total particulate matter (TPM) smoke emissions coefficient ($C_{e}$) in Figure 3. Data for the plume subsets and the fires contained within the plume polygon depicted in Figure 2 are shown.

Sample ID	Date (2015)	FRE (10^7^ MJ)	TPM (10^7^ g)	Mean Plume AOD	Plume Area (10^8^ m^2^)	Plume Length (km)	Plume Velocity (ms^−1^)	Time (s)	Landcover Concession Type
1	07/06	0.98	16.6	0.65	10.9	35.9	13.1	2753	oil palm
2	08/07	0.167	2.1	0.38	2.39	26.4	10.3	2553	none
3	08/07	0.07	5.7	0.57	4.34	37.6	7.6	4951	none
4	08/07	0.80	9.9	0.66	6.38	25.1	4.7	5297	none
5	09/11	0.32	10.3	1.94	2.43	21.5	10.6	2030	fibre
6	09/22	0.57	19.9	2.02	3.67	30.6	11.5	2665	none
7	09/23	1.11	26.0	2.11	5.24	32.4	21.4	1512	none
8	09/23	0.32	7.4	1.78	1.99	23.6	11.1	2137	fibre
9	09/24	0.35	3.5	0.63	2.43	22.5	10.3	2186	fibre
10	10/03	1.28	17.8	1.82	4.53	23.9	12.3	1937	fibre
11	10/04	1.62	25.0	2.41	5.04	26.3	10.5	2497	fibre
12	10/20	1.12	14.4	2.08	4.23	13.3	21.5	618	fibre
13	10/20	0.07	0.6	0.15	1.89	22.9	14.1	1634	none

**Table 2:** Existing peatland and tropical forest fire TPM smoke emissions coefficients (*C_e_*) linking TPM emissions to FRE release, for comparison to that derived herein (Figure 3).

Source	Peatlands (g·MJ^−1^)	Tropical Forests (g·MJ^−1^)
[27]	69.3	11.3
[37]	52.4	30.0
[44]	-	15–32

**Figure A1:**
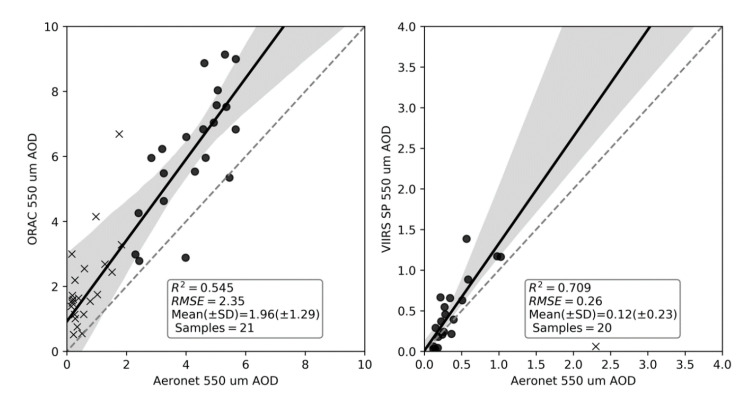
Intercomparison between collocated AOD retrievals made from VIIRS using ORAC [52] and the official VIIRS IP AOD product [49], both conducted at 550 nm and for the set of SE Asian fires focused on herein. ORAC was able to retrieve AODs significantly higher than those present in the VIIRS IP AOD product, so the scales of the two graphs are different. The OLS linear best fit through each set of data is shown in both cases, and at left the crosses relate to points with AOD < 2.0.

**Figure A3:**
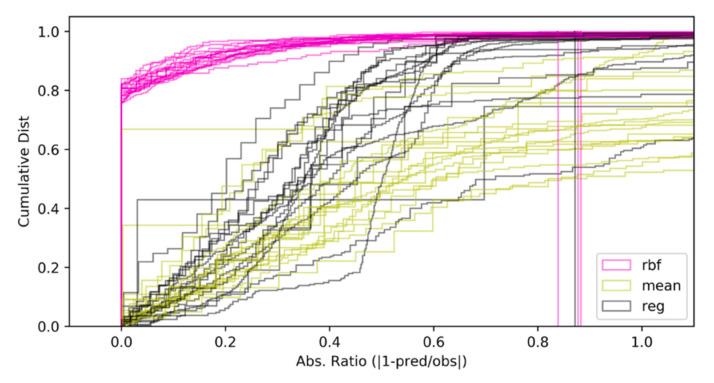
Assessment of three different approaches (radial basis function (RBF), mean value, and regression) for generating AOD estimates for missing pixels. The *x*-axis shows the error function evaluated for each approach, where *pred* is the predicted AOD for a missing pixel and *obs* is the observed AOD in that pixel. The radial basis function (RBF) performs best overall for the thirteen plumes evaluated, with typically ~80% of the refilled pixels evaluated in each plume having an error near zero.

**Figure A6:**
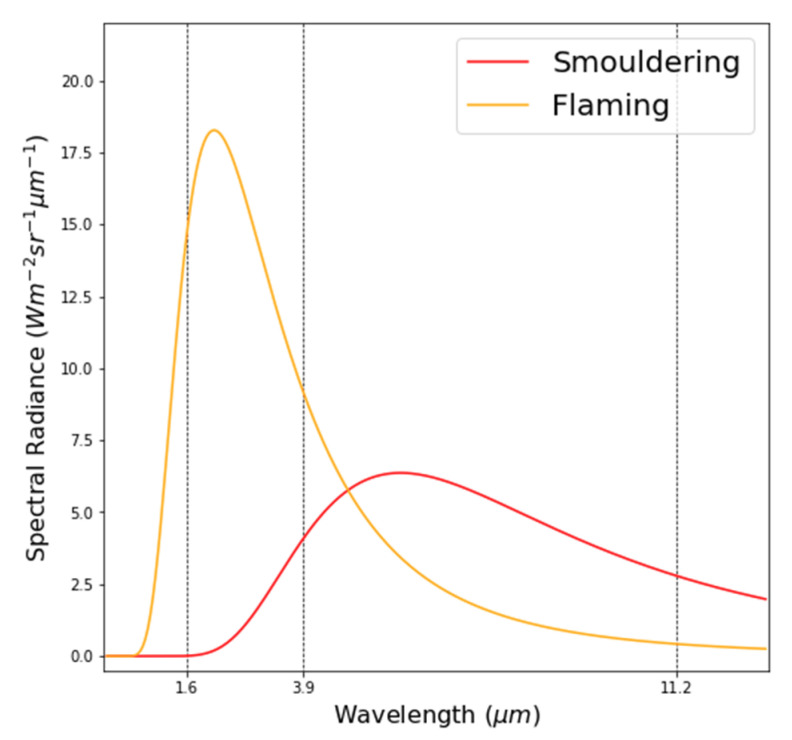
Planck curves for blackbodies at temperatures typical for flaming and smouldering combustion.

**Figure A7:**
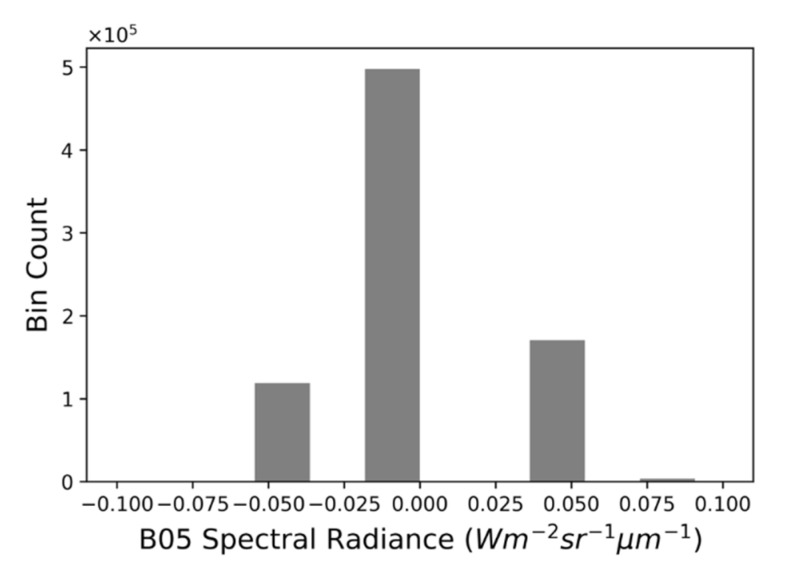
Spectral radiance distribution of nonthermal anomaly containing AHI B05 (1.6 µm) pixels for 25 September 2015 for the region shown in Figure A8.

**Figure A8:**
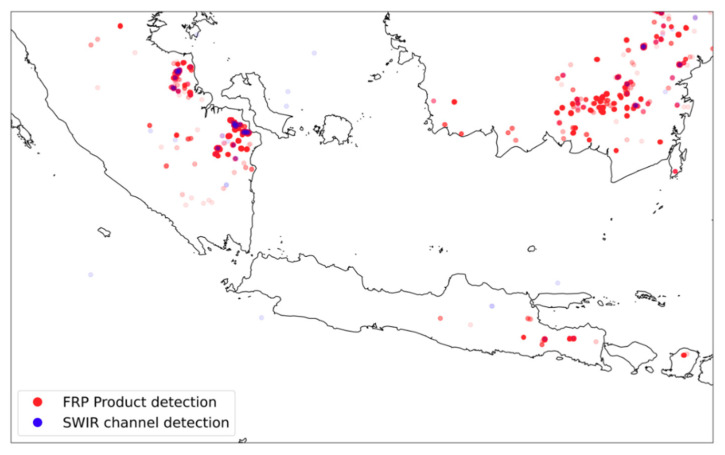
In red are all hotspots detected by the AHI FRP product on 25 September 2015 and in blue are all AHI SWIR pixels with a spectral radiance value >0.1 W m^2^ sr^−1^ µm^−1^.

**Figure A9:**
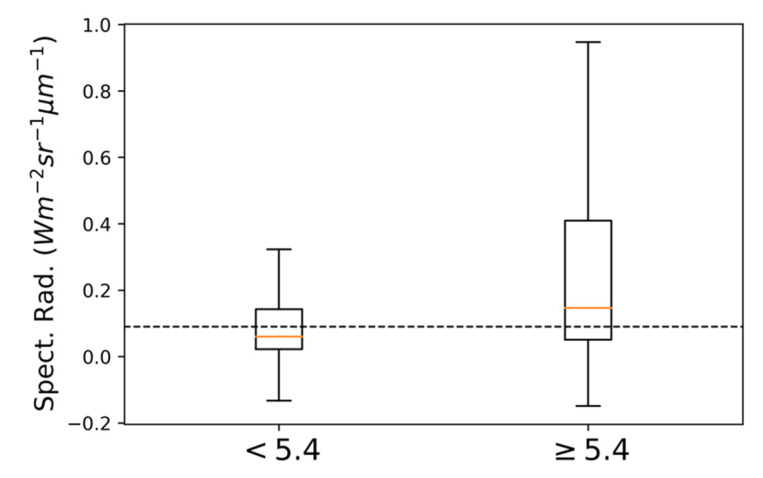
Boxplots of collocated SWIR spectral radiances for all AHI FRP product thermal anomaly containing pixels shown in Figure A8 binned based on whether the pixel meets the MWIR/LWIR spectral ratio threshold of 5.4 W m^2^ sr^−1^ µm^−1^ (W m^2^ sr^−1^ µm^−1^)^−1^ or not. The boxplots follow the same conventions defined in Figure 5.

## Appendix A

To assess the satellite-derived AODs used herein, we made use of the AERONET database, which was reprocessed in 2018 with the AERONET Version 3 algorithm. This provided a number of improvements to the cloud screening and instrument anomaly detection processes [65], including a new very high AOD restoration step that retained previously excluded cases of high aerosol loading typically associated with biomass burning plumes [66]. The uncertainty in the V3 AOD is ~0.01 (±0.015) optical depths at visible wavelengths [65], very similar to that in the V2 algorithm. As shown in Figure A1, a number of AERONET stations are located in the SE Asian region of interest, and for the period August through October 2015 those located at Jambi, Kuching, Makassar, Palangkaraya, Pontianak, and Singapore were examined for the occurrence of overlying smoke and clouds (manually assessed using VIIRS true colour composites). When an overlying smoke plume was found to coincide with an AERONET station location, the AERONET database was queried for a temporally collocated Level 2.0 (cloud screened and quality assured) AOD sample, with a time difference between the VIIRS overpass and the AERONET sampling of up to ±30 min permitted. To account for the differences in spatial sampling characteristics between AERONET and the VIIRS imagery, all AERONET samples within ±30 min of the VIIRS overpass were averaged and the VIIRS AODs retrieved with the ORAC optimal estimation aerosol and cloud retrieval algorithm [67] and from the official VIIR IP AOD product [49] were averaged over a 10 km^2^ window centred on the station location. For a spatiotemporal collocation to be included in the assessment, at least three AERONET and five “good” quality VIIRS AOD retrievals (at 500 and 650 nm) had to be available for averaging; for ORAC this is indicated by a retrieval cost of <10 and for the IP data a retrieval flag of 0.

This procedure identified 50 suitable samples across the AERONET station subset under consideration, comprising 22 samples with smoke features with optical depths ≥2.0 and 28 samples with optical depths <2.0. As AERONET stations do not retrieve AOD at 550 nm, it is interpolated from the measurement at 650 nm using the wavelength specific angstrom exponent. From the VIIRS observations, a total of 43 (out of 50) collocated ORAC retrievals were available for intercomparison to the AERONET station AODs and these are shown in Figure A1. The collocated retrievals have AODs ranging from ~0.1 to ~5.0 for AERONET and ~0.5 to ~9.0 for ORAC, and for the samples with AERONET optical depths <2.0 a mean AOD difference of 1.41 ± 1.06 is observed with the ORAC retrievals from VIIRS, whereas for the samples with AERONET optical depths ≥2.0 differences were 1.96 ± 1.29. There appears significantly more spread for the data obtained in less optically thick smoke conditions (Figure A1), agreeing with the fact that ORAC AOD retrievals for single view sensors such as VIIRS retain significant sensitivity to the underlying land surface reflectance in such conditions, introducing more uncertainty [52]. Both optically thick and thin observations show a positive bias of the ORAC-derived AODs compared to AERONET, and the cause seems likely to be differences between the optical properties of the aerosols assumed in ORAC and the true optical properties of the smoke. However, the observed bias is systematic and adjustable using a simple linear correction (slope: 1.25; intercept 0.92) (Figure A1). This adjustment is applied to all ORAC-derived AODs used herein.

For the IP aerosol product, a total of 20 (out of 28) collocated high quality retrievals were available for intercomparison, also shown in Figure A1. A small bias of 0.12 ± 0.23 is observed between these retrievals and those of AERONET, indicating that for the smoke conditions focused on here with the VIIRS IP product the AOD estimates are generally sound, supporting the findings of more extensive assessments reported elsewhere [68,69]. However, the IP product cannot be used to assess very high AOD values, which is the reason that the ORAC-retrievals of AOD are required for such situations. All but one of the IP AODs are associated with AERONET observations of less than 2.0 optical depths; however, eight (~29%) of the AERONET samples with AODs of <2.0 are not retrieved with high quality by the IP algorithm, potentially indicating possible coverage issues.

## Appendix B

The openCV2 optical flow parameters used to estimate motion between Himawari-8 image acquisitions are pyr_scale = 0.5, levels = 1, winsize = 5, iterations = 7, poly_n = 7, poly_sigma = 1.5, and flags = cv2.OPTFLOW_FARNEBACK_GAUSSIAN.

## Appendix C

Three different AOD interpolation approaches were assessed for their performance in providing estimates for missing pixels in the AOD products. The first approach assessed is the most basic and involves replacing missing data points with a mean value derived from the entire plume. The second replaces missing points using estimates generated from a multiple linear regression that associates spectral radiances in the VIIRS M3, M4, and M5 bands with the observed aerosol optical depth. The final approach assessed is radial basis function (RBF) interpolation. To evaluate the three approaches, all thirteen plumes in this study had 25% of their valid AOD retrievals (from the merged ORAC and IP AOD product generated using the approach described in Section 3.3) removed, and values for those locations were refilled using the three approaches considered herein. In Figure A3, the results for each plume and interpolation method are shown, with the evaluation metric being the cumulative distribution of 1− |pred/obs| for all refilled pixels in the plume, where *pred* is the predicted AOD and *obs* is the observed AOD. The RBF approach clearly outperforms the alternative methods, with nearly 80% of the refilled data points across all plumes having an interpolation error of near 0, and ~90% of the interpolated pixels have an error of <20%. The alternative methods do not perform nearly as well, hence the selection of the RBF interpolation approach to replace missing AOD values in this study.

## Appendix E

To assess the suitability of the MWIR/LWIR spectral ratio defined in Section 4.2, night-time collocated SWIR observations at 1.6 µm can be used. Shown in Figure A6 are two Planck curves, one assuming 5% of a unit pixel combusting at 500 K (i.e., smouldering) and the other 0.01 of a unit pixel combusting at 1300 K (i.e., flaming). It is apparent that at 500 K the spectral radiance produced at 1.6 µm is close to zero, whereas there is significant spectral radiance at the flaming combustion temperature. At night, in the absence of reflected solar radiation, 1.6 µm hotspot pixels raised above the background signal therefore must contain flaming activity. The distribution of night-time background signal (i.e., instrument noise) for AHI 1.6 µm (B5) imagery (12:00:00 UTC to 20:00:00 UTC) over the study region shown on Figure A8 for 25 September 2015 is shown in Figure A7. As shown in the distribution, it can be assumed that any night-time SWIR pixel with spectral radiances above 0.1 W m^2^ sr^−1^ µm^−1^ contains flaming activity. In Figure A8, all thermal anomalies detected by the AHI FRP product on 25 September are shown and overlain are all AHI night-time (12:00:00 UTC to 20:00:00 UTC) 1.6 µm SWIR detections that exceed the 0.1 W m^2^ sr^−1^ µm^−1^ threshold. It is apparent that many thermal anomalies detected by the AHI FRP product are not detected in the SWIR imagery, indicating the lack of any intense flaming activity in these fires. To assess the suitability of the MWIR/LWIR spectral ratio, the observed night-time AHI 1.6 µm spectral radiances can therefore be used, with the expectation that for MWIR/LWIR spectral ratios of <5.4 the observed 1.6 µm radiances should be near the sensor noise floor (i.e., <0.1 W m^2^ sr^−1^ µm^−1^) and that for MWIR/LWIR spectral ratios of ≥5.4 the observed 1.6 µm spectral radiances should be above the noise floor (i.e., ≥0.1 W m^2^ sr^−1^ µm^−1^). This analysis is shown in Figure A9, and it can be seen that for MWIR/LWIR spectral ratios of <5.4 the observed 1.6 µm radiances are found to be less than 0.1 W m^2^ sr^−1^ µm^−1^, with a median value of 0.06 W m^2^ sr^−1^ µm^−1^ and for spectral ratios of ≥5.4 the 1.6 µm radiances are typically greater than 0.1 W m^2^ sr^−1^ µm^−1^ with a median value of 0.15 W m^2^ sr^−1^ µm^−1^. This demonstrates that MWIR/LWIR spectral ratio values of >5.4 are typically associated with thermal anomalies that produce detectable signals at 1.6 µm at night, which is indicative of flaming combustion.
